# Salivary Levels of Hemoglobin for Screening Periodontal Disease: A Systematic Review

**DOI:** 10.1155/2018/2541204

**Published:** 2018-03-20

**Authors:** Yoshiaki Nomura, Ayako Okada, Yoh Tamaki, Hiroko Miura

**Affiliations:** ^1^Department of Translational Research, School of Dental Medicine, Tsurumi University, 2-1-3 Tsurumi, Tsurumi-ku, Yokohama 230-8501, Japan; ^2^Department of Health and Welfare Services, National Institute of Public Health, 2-3-6 Minami, Wako-shi, Saitama 351-0197, Japan; ^3^Department of International Health and Collaboration, National Institute of Public Health, 2-3-6 Minami, Wako-shi, Saitama 351-0197, Japan

## Abstract

Periodontal disease is a common inflammatory disease. It affects about 20–50% of global population in both developed and developing countries. Early detection of slight changes of periodontal tissue plays an important role in prevention of onset and progression of periodontal disease. Hence, there is a need of a screening test to assess periodontal tissue for health check-ups. Salivary levels hemoglobin (Hb) has been proposed to assess the conditions of the inflammation of gingiva. The aim of this systematic review was to evaluate and summarize critically the current evidences for Hb as periodontal screening test. We performed a literature search of report published using PubMed databases. A total of 55 articles were retrieved and 16 were selected. Our review focuses on corelation coefficient with periodontal clinical parameters or sensitivity and specificity. As a result, fourteen studies calculated sensitivity and specificity of Hb. Six studies measured salivary levels hemoglobin at laboratory: three studies used polyclonal antibody reactions and other studies used colorimetric tests. Eight studies used paper strip method: 4 studies used monoclonal antibody reaction and 4 studies used colorimetric tests. Youden's indexes by antibody reaction were better than those of colorimetric methods. Evidences are described above and further studies are necessary to set the cut off values stratified by gender, age and number of remaining teeth.

## 1. Introduction

Periodontal diseases comprise a wide range of inflammatory conditions that affect the supporting structures of the teeth, which could lead to tooth loss and contribute to systemic inflammation. Periodontal disease is prevalent both in developed and developing countries and affect about 20–50% of global population. High prevalence of periodontal disease in adolescents, adults, and older individuals makes it a public health concern [1]. Early detection and treatment is important for periodontal disease to prevent the progression and to keep up systemic health.

In Japan, health check-up systems are established at each life stage, and the government regulates these check-up systems. In the health check-up system, oral examinations were included for pregnant women and infants at the age of 18 months and 36 months as maternal and child health, from elementary school children to high school student as school health. After that age, municipalities conduct dental check-ups for the resident at the age of 40, 50, and 60 years.

For laborers, company conducts annual medical check-ups for the prevention of noncommunicable disease. However, in this check-up system, oral examination is not included. Even though, periodontal disease symptoms appear at adults, and older individuals, the oral examinations at these ages are limited.

One of the obstacles for the popularization of periodontal check-ups is the cost. Dental professional is indispensable for the conventional oral examinations, and number of the subjects is limited for the oral examination. There is a pressing need for a reliable, low-cost method of assessing the gingival and periodontal status of large population groups.

Even the subjects with periodontal awareness did not visit dental clinics. Self-diagnosis of “Treatment not necessary” was found to be the common barrier for regular dental attendance [2]. Therefore, the results of screening tests may help for the regular dental attendance.

Salivary levels hemoglobin (Hb) is one of the candidates to assess the periodontal conditions without dental professionals and low-cost method alternative for oral examinations. Several advanced biomarkers had been proposed for assessing periodontal conditions; however, measuring these markers is not approved for extracorporeal diagnostic agents even for the blood tests. Especially, in Japan, only salivary levels hemoglobin i approved by the Pharmaceutical Affairs Law as extracorporeal diagnostic agents for assessing periodontal conditions.

In addition, saliva tests of Hb are an application of the fecal occult blood. It can detect subtle bleeding form gingival tissue. It may lead to early detection. If saliva tests can incorporate in medical check-up system, it may be the help of health promotion including oral health.

This study describes an evaluation of previously published article about salivary levels of hemoglobin as screening test for periodontal disease.

## 2. Materials and Methods

### 2.1. Search Strategy

Electronic database, PubMed was searched for eligible studies. Abstracts were reviewed, and full-text articles were inspected for inclusion criteria. The reference lists from reviewed studies were also examined to seek additional sources. The country of origin, study design, population, periodontal index used, method of measurement, sensitivity, and specificity were reviewed for each study.

Key word search terms using medical subject headings and text words included. Search details were as follows: (“hemoglobins” [MeSH Terms] OR “hemoglobins” [All Fields]) AND (“gingivitis” [MeSH Terms] OR “gingivitis” [All Fields]) OR (“periodontitis” [MeSH Terms] OR “periodontitis” [All Fields]) AND (“screening” [All Fields] OR “mass screening” [MeSH Terms] OR (“mass” [All Fields] AND “screening” [All Fields]) OR “mass screening” [All Fields] OR “screening” [All Fields]) AND (“saliva” [All Fields])

Studies were included if they tested human subjects for the screening of periodontal disease by salivary levels of Hb and were written in English.

### 2.2. Study Selection

An initial search for studies using saliva for the evaluation of periodontitis generated 55 articles. During the preliminary analysis, 39 studies were excluded because they reported HbA1c, not measuring salivary levels of Hb. Finally, 16 articles were reviewed. The investigators then independently evaluated 16 articles.

### 2.3. Outcome Measures

Two outcome measures were used. The effectiveness of saliva tests for the screening of periodontal disease was evaluated by mainly two methods: sensitivity and specificity or corelation coefficient with periodontal clinical parameters. From the sensitivity and specificity, positive likelihood ratio (sensitivity/(1 − specificity)) and negative likelihood ratio (specificity/(1 − sensitivity)) and Youden's index (sensitivity + specificity − 1) were calculated.

## 3. Results

### 3.1. Study Characteristics

One study was conducted to compare the performance of two commercially available products for measuring salivary levels of Hb [3]. One study calculated the corelation coefficient between salivary levels of Hb and periodontal index [4]. Other 14 studies calculated sensitivity and specificity for the screening of periodontal disease by salivary levels of Hb in Table 1 [5–18].  Table 2 showed the characteristics of the included the studies. Among these 14 studies, 6 studies measured Hb at laboratory by commercially available products like Hb in faeces or urine [9, 13–15, 17, 18]. Three used polyclonal antibody reaction and 3 other studies used colorimetric tests. Seven studies used paper strip method. Three studies used Perioscreen SUNSTAR (SUNSTAR, Osaka, Japan), which used monoclonal antibody against human Hb and 4 studies used colorimetric tests.

Periodontal indexes used as the gold standard varied between studies. One study used the criteria by the American Academy of Periodontology. Four studies used the Community Periodontal Index. One study used PD ≥ 4 mm. One study used progression of CAL > 3 mm. This study is the only one longitudinal study, and other studies were all cross-sectional studies.

Youden's indexes of Hb measured in laboratory by polyclonal antibody reaction ranged from 0.08 to 0.52. Those by colorimetric method ranged from 0 to 0.08. Youden's indexes of Perioscreen SUNSTAR were 0.24 to 0.50. Those of other paper strip methods were 0.17 to 0.57. Six studies used adjustment by statistical modeling.

## 4. Discussion

We reviewed studies for the screening periodontal disease by salivary levels of Hb. The measuring method of Hb, periodontal indexes used as a gold standard varied between studies. There are two methods for measuring Hb: colorimetric test and antibody reactions. The colorimetric tests are based on the reaction that the hemoglobin decomposes the hydrogen peroxide with the liberation of oxygen, oxidizing the orthotolidine to a blue colored derivative. For measuring Hb, the specificity of colorimetric tests is not superior to antibody reaction because it reacts with Hb of other animals contained in food. In general, the colorimetric method has been used for measuring Hb in urine. It is quite unlikely to contaminate blood form other animals in urine. Antibody reactions are used to detect Hb in fecal blood because, in feces, there may be remained various Hb from other animals by food. In this respect, antibody reactions are more suitable to detect Hb from oral cavity. In fact, Youden's indexes of antibody reaction measured in laboratory were better than that of the colorimetric method. The commercial products approved by Japanese Pharmaceutical Affairs Law to measure the Hb in saliva are all used antibody reactions including paper strip method (Perioscreen SUNSTAR).

The periodontal indexes used varied. It is likely that pure saliva from saliva ducts contains lilted or no Hb except for salivary gland inflammation. The Hb in saliva may be derived mainly from the bleeding of the periodontal tissue. Bleeding on probing (BOP) is the index that evaluates the bleeding from gum. When BOP was used as the gold standard of the screening, high sensitivity and specificity may be easily obtained when compared with other periodontal indexes. In fact, Youden's indexes by BOP ranged from 0.19 to 0.59. The index by PD 4 mm was 0.08 and that by American Academy of Periodontology was 0.52. The tooth that accompanied deep periodontal pockets does not always accompany bleeding from the periodontal pocket. Three studies used Community Periodontal Index (CPI) as a gold standard. Originally, CPI is a method for the survey proposed by the World Health Organization. It measured the sextant of representative 6 teeth. This method was sometimes used for the screening of periodontal disease. However, both saliva tests and CPI are a method for the screening of periodontal disease. Therefore, applying CPI as a gold standard for the saliva tests is not suitable. More precise definition of periodontal disease should be used for the standard.

For measuring Hb in laboratory (stimulated saliva) and Perioscreen SUNSTAR (resting saliva), method of saliva sampling is established because these reagents are approved by the Japanese Pharmaceutical Affairs Law. To obtain the approval, reporting the performance of the reagent, standardized measuring method, and established sampling method are indispensable.

Studies had shown that by the adjustment by statistical model, the sensitivity and specificity were improved. In the check-ups, to apply statistical model for every examination is insubstantial. These studies suggest that cut off values were in part dependent on the age, gender, number of remaining teeth, or subjective symptom of the subjects. Therefore, cut off values should be set separately by age, gender, or number of remaining teeth.

## 5. Conclusions

Saliva tests can be applied by nonprofessional personnel. Further study is necessary to set the cut off values stratified by gender, age, and number of remaining teeth.

## Figures and Tables

**Table 1:** Selected articles.

Author(s) and year published	Title of journal	Journal	Number of subjects	Results
Burt et al. 1978 [5]	Saliva-based colorimetric test as an index of gingival inflammation in epidemiologic studies	Community Dentistry and Oral Epidemiology	253	Colorimetric test may be a valid, reliable means of detecting major differences in the prevalence of gingival inflammation in most adult populations, although having little, if any, useful application among children at the mixed dentition stage.
Abbott and Caffesse 1978 [6]	The reliability of a colorimetric test in determining gingival inflammation	Journal of Periodontology	81	The G index chemical analysis may be a useful diagnostic aid for detecting the presence or absence of gingival inflammation in dental practice.
Kopczyk et al. 1995 [7]	The feasibility and reliability of using a home screening test to detect gingival inflammation	Journal of Periodontology	50	The test should be used as a home screening tool rather than a diagnostic aid. Patients who test positive for occult blood should seek advice from a dental professional.
Nomura et al. 2006 [8]	Screening of periodontitis with salivary enzyme tests	Journal of Oral Science	187	LDH level had the highest sensitivity and specificity (sensitivity, 0.66; specificity 0.67).
Kugahara et al. 2008 [9]	Screening for periodontitis in pregnant women with salivary enzymes	Journal of Obstetrics and Gynaecology Research	221	Combining LDH, ALP, and occult blood showed the highest diagnostic performance, with a sensitivity value of 0.90, specificity value of 0.62, positive predictive value of 0.18, and negative predictive value of 0.98.
Ohshima et al. 2009 [10]	Comparison of periodontal health status and oral health behavior between Japanese and Chinese dental students	Journal of Oral Science	92	Pain in gum, swollen gum, and bleeding gum had statistically significant corelation with the result of Perioscreen.
Shimazaki et al. 2011 [11]	Effectiveness of the salivary occult blood test as a screening method for periodontal status	Journal of Periodontology	1998	The sensitivity and specificity of the salivary occult blood test in screening for poor periodontal status were 0.72 and 0.52, respectively. In a multivariate logistic regression analysis, the results were significantly associated with the proportion of teeth with BOP and the proportion of teeth with PD ≥ 4 mm, independent of age, sex, use of antihypertensive medication, use of antidiabetic medication or insulin therapy, and the number of decayed or filled teeth.
Pham et al. 2011 [12]	Periodontal disease and related factors among Vietnamese dental patients	Oral Health & Preventive Dentistry	243	The perioscreen test showed moderate sensitivity (0.752) and specificity (0.746) to periodontal disease. The binary logistic regression analyses indicated that older subjects (OR = 2.5), or those who did not frequently visit a dentist (OR = 4.1), brushed their teeth only once a day (OR = 2.5), did not use dental floss (OR = 2.9), were past smokers (OR = 3.1), current smokers (OR = 4.1), or had positive BANA test results (OR = 12.0) were more likely to have periodontal disease.
Nomura et al. 2012 [13]	Screening for periodontal diseases using salivary lactate dehydrogenase, hemoglobin level, and statistical modeling	Journal of Dental Sciences	101	By the statistical modeling, sensitivity and specificity were improved for the screening of periodontal disease by the salivary levels of hemoglobin and LD.

Nomura et al. 2012 [14]	Salivary biomarkers for predicting the progression of chronic periodontitis	Archives of Oral Biology	85	Salivary ALT level and the *P. gingivalis* ratio may be potential indicators for the progression of periodontitis. Such a salivary test could be a useful diagnostic tool for predicting periodontal disease progression.
Nam et al. 2015 [15]	Validity of screening methods for periodontitis using salivary hemoglobin level and self-report questionnaires in people with disabilities	Journal of Periodontology	195	The salivary hemoglobin level, self-report questionnaire, and the combined method demonstrated screening potential that could predict the population prevalence of CPI 3 or CPI 4.
Reed et al. 2015 [16]	Feasibility study of a salivary occult blood test to correlate with periodontal measures as indicators of periodontal inflammation in a population of pregnant women	Journal of Oral Science	23	Pearson correlation coefficient with the percent of sites with BOP was statistically significant (0.301, *P* value 0.0469), and with BOP as the sum of sites with bleeding on probing was 0.280, *P* value 0.0647.
Nomura et al. 2016 [17]	A new screening method for periodontitis: an alternative to the community periodontal index	BMC Oral Health	92	The sensitivity and specificity for hemoglobin levels were, respectively, 0.722 and 0.711, for lactate dehydrogenase levels. Combining these two tests, when samples tested positive for both hemoglobin and lactate dehydrogenase, the positive predictive value was 91.7%.
Maeng et al. 2016 [18]	Diagnostic accuracy of a combination of salivary hemoglobin levels, self-report questionnaires, and age in periodontitis screening	Journal of Periodontal & Implant Science	202	The combination of salivary hemoglobin levels and self-report questionnaires was shown to be a valuable screening method for detecting periodontitis.

**Table 2:** Characteristics of the included the studies.

References	Subjects	Exclusion criteria	Definition of periodontitis	Measuring method		Saliva sample	Adjustment	Results				
				Reaction	Products			Sensitivity	Specificity	Positive likelihood ratio	Negative likelihood ratio	Youden's index
*Laboratory examination*												
Kugahara et al. [9]	221 pregnant women (mean age: 30 ± 4 years)	Current smokers and subjects with not sufficient saliva were excluded	Periodontitis (CPITN 3 and 4)	Colorimetric test	Salivastar-Bld	—		0.37	0.91	4.11	1.44	0.28
							Combination with LDH	0.68	0.74	2.62	2.31	0.42
Nomura et al. [13]	187 subjects (mean age: 37.2 ± 9.6 years)	—	PD ≥ 4 mm	Colorimetric test	—	Stimulated saliva	—	0.27	0.81	1.42	1.11	0.08
Nomura et al. [14]	85 patients with chronic periodontitis	Clinical attachment level (CAL) of 3 mm in at least three subsequent assessments	Progression of CAL ≥ 3 mm	Colorimetric test	—	Stimulated saliva	—	0.32	0.68	1.00	1.00	0.00
Nam et al. [15]	The participants were 195 patients with disabilities (aged >18 years)	—	CPI scores of 3-4	Polyclonal antibody reaction	OC-HEMODIA AUTO S	Stimulated saliva	—	0.41	0.77	1.78	1.31	0.18
			CPI scores of 4				—	0.53	0.75	2.12	1.60	0.28
			CPI scores of 3-4				With questionnaire	0.58	0.76	2.42	1.81	0.34
			CPI scores of 4				With questionnaire	0.73	0.74	2.81	2.74	0.47
			CPI scores of 3-4				With questionnaire and age	0.70	0.76	2.92	2.53	0.46
			CPI scores of 4				With questionnaire and age	0.71	0.80	3.55	2.76	0.51
Nomura et al. [17]	92 subjects (mean age: 50.03 years)	Patients older than 20 years who had more than 20 teeth remaining were included	Center for Disease Control and Prevention in partnership with the American Academy of Periodontology.	Polyclonal antibody reaction	OC-HEMODIA AUTO S	Stimulated saliva	—	0.76	0.76	3.20	3.17	0.52

Maeng et al. [18]	202 subjects (age: 20 to 79 years)	Pregnant, had undergone a periodontal operation within the past month, or had an injury accompanied by oral bleeding, such as a wound or ulcer	CPI scores of 3-4	Polyclonal antibody reaction	OC-HEMODIA AUTO S	Stimulated saliva	—	0.71	0.56	1.61	1.93	0.27
			CPI scores of 4				—	0.60	0.72	2.14	1.80	0.32
			CPI scores of 3-4				With questionnaire	0.71	0.68	2.25	2.38	0.40
			CPI scores of 4				With questionnaire	0.65	0.77	2.79	2.17	0.00
*Paper strip*												
Burt et al. [5]	136 school children, 52 adult dental hygene school students (age: 24 to 32) and 65 adults in correctional Institution (age: 18 to 66)	—	GI score (0–0.6, <1.2, <1.21, <0.2, <0.4, <0.41)	Colorimetric test	G index	Resting saliva						
			GI > 0.6				School children	0.42	0.64	1.17	1.10	0.06
			GI > 1.2					0.50	0.63	1.35	1.26	0.13
			GI > 0.6				Innate correctional Institution	0.65	0.76	2.71	2.17	0.41
			GI > 1.2					0.67	0.53	1.43	1.61	0.20
			GI > 0.2				dental hygiene student	0.25	0.64	0.69	0.85	−0.11
			GI > 0.4					0.16	0.67	0.48	0.80	−0.17
Abbott and Caffesse [6]	81 subjects (age: 15 to 60 years)		GI > 0	Colorimetric test	G index	Before any clinical examination	—	0.83	0.63	2.24	3.71	0.46
			GI > 1					1.00	0.46	1.85	—	0.46
			GI > 2					1.00	0.29	1.41	—	0.29
Kopczyk et al. [7]	50 patients with less than 20 teeth 27 (age: 27 to 72 years)	Using aspirin or nonsteroidal anti-inflammatory drugs. Those with bleeding disorders, ulcerating oral lesions, or medical contraindications	BOP > 30%	Colorimetric test	Seracult	Resting saliva before and after tooth brush	Before tooth brush	0.19	1.00	—	1.23	0.19
			BOP > 50%					0.56	0.97	18.67	2.20	0.53
			BOP > 30%				After tooth brush	0.75	0.82	4.17	3.28	0.57
			BOP > 50%					0.85	0.66	2.50	4.40	0.51

Nomura et al. [8]	187 subjects (mean age 37.2 ± 9.6 years)	—	PD ≥ 4 mm	Colorimetric test	—	Stimulated saliva	—	0.27	0.81	1.42	1.11	0.08
Ohshima et al. [10]	92 dental school students (mean age: 22.1 years)	—	—	Monoclonal antibody reaction	Perioscreen SUNSTAR	—	—	—	—	—	—	—
Shimazaki et al. [11]	1998 subjects with less than 20 teeth (age: 40 to 79)	—	BOP ≥ 15% or at least one PD ≥ 4 mm	Monoclonal antibody reaction	Perioscreen SUNSTAR	Rinse 3 ml distilled water for 10 sec 8:00 am to 11:00 am before oral examination	—	0.69	0.55	1.53	1.77	0.24
Pham et al. [12]	243 subjects	—	—	Monoclonal antibody reaction	Perioscreen SUNSTAR	—	—	0.75	0.75	2.96	3.01	0.50
Reed et al. [16]	23 pregnant women (age: 18 to 45 years)	Preexisting parathyroid disease or uncontrolled thyroid disease, multiple fetuses (e.g., twins and triplets), preexisting sickle cell disease (not trait only), sarcoidosis, Crohn's disease, or ulcerative colitis	—	Monoclonal antibody reaction	Perioscreen SUNSTAR	—	—	—	—	—	—	—

## References

[1] Nazir M. A. (2017). Prevalence of periodontal disease, its association with systemic diseases and prevention. *International Journal of Health Sciences*.

[2] Taani D. Q. (2002). Periodontal awareness and knowledge, and pattern of dental attendance among adults in Jordan. *International Dental Journal*.

[3] Okada A., Nomura Y., Sogabe K. (2017). Comparison of salivary hemoglobin measurements for periodontitis screening. *Journal of Oral Science*.

[4] An Y. B., He L., Meng H. X. (2010). Relationship between salivary occult blood and level of volatile sulphur compounds in oral cavity. *Zhonghua Kou Qiang Yi Xue Za Zhi*.

[5] Burt B. A., Roder D. M., Cecil J. C. (1978). Saliva-based colorimetric test as an index of gingival inflammation in epidemiologic studies. *Community Dentistry and Oral Epidemiology*.

[6] Abbott B. H., Caffesse R. G. (1978). The reliability of a colorimetric test in determining gingival inflammation. *Journal of Periodontology*.

[7] Kopczyk R. A., Graham R., Abrams H. (1995). The feasibility and reliability of using a home screening test to detect gingival inflammation. *Journal of Periodontology*.

[8] Nomura Y., Tamaki Y., Tanaka T. (2006). Screening of periodontitis with salivary enzyme tests. *Journal of Oral Science*.

[9] Kugahara T., Shosenji Y., Ohashi K. (2008). Screening for periodontitis in pregnant women with salivary enzymes. *Journal of Obstetrics and Gynaecology Research*.

[10] Ohshima M., Zhu L., Yamaguchi Y. (2009). Comparison of periodontal health status and oral health behavior between Japanese and Chinese dental students. *Journal of Oral Science*.

[11] Shimazaki Y., Akifusa S., Takeshita T. (2011). Effectiveness of the salivary occult blood test as a screening method for periodontal status. *Journal of Periodontology*.

[12] Pham T. A., Ueno M., Shinada K. (2011). Periodontal disease and related factors among Vietnamese dental patients. *Oral Health & Preventive Dentistry*.

[13] Nomura Y., Tamaki Y., Eto A. (2012). Screening for periodontal diseases using salivary lactate dehydrogenase, hemoglobin level, and statistical modeling. *Journal of Dental Sciences*.

[14] Nomura Y., Shimada Y., Hanada N. (2012). Salivary biomarkers for predicting the progression of chronic periodontitis. *Archives of Oral Biology*.

[15] Nam S. H., Jung H., Kang S. M. (2015). Validity of screening methods for periodontitis using salivary hemoglobin level and self-report questionnaires in people with disabilities. *Journal of Periodontology*.

[16] Reed S. G., Manz M. C., Snipe S. M. (2015). Feasibility study of a salivary occult blood test to correlate with periodontal measures as indicators of periodontal inflammation in a population of pregnant women. *Journal of Oral Science*.

[17] Nomura Y., Okada A., Kakuta E. (2016). A new screening method for periodontitis: an alternative to the community periodontal index. *BMC Oral Health*.

[18] Maeng Y. J., Kim B. R., Jung H. I. (2016). Diagnostic accuracy of a combination of salivary hemoglobin levels, self-report questionnaires, and age in periodontitis screening. *Journal of Periodontal & Implant Science*.

